# Immune-inflammatory markers and arterial stiffness indexes in subjects with acute ischemic stroke with and without metabolic syndrome

**DOI:** 10.1186/1758-5996-6-28

**Published:** 2014-02-27

**Authors:** Antonino Tuttolomondo, Rosaria Pecoraro, Domenico Di Raimondo, Riccardo Di Sciacca, Baldassare Canino, Valentina Arnao, Carmelo Buttà, Vittoriano Della Corte, Carlo Maida, Giuseppe Licata, Antonio Pinto

**Affiliations:** 1 Dipartimento Biomedico di Medicina Interna e Specialistica, Università degli Studi di Palermo, P.zza delle Cliniche n.2, 90127 Palermo, Italy; 2 Dipartimento di Biomedicina Sperimentale e Neuroscienze Cliniche, Università degli Studi di Palermo, Palermo, Italy

**Keywords:** Stroke, Arterial stiffness, PWV, Aix, Cytokines

## Abstract

**Objective:**

The aim of our study was to evaluate the associations between arterial stiffness indexes and immune-inflammatory markers in subjects with acute ischemic stroke with and without metabolic syndrome.

**Materials/Methods:**

We enrolled 130 patients with acute ischemic stroke and metabolic syndrome, 127 patients with acute ischemic stroke without metabolic syndrome and 120 control subjects without acute stroke. Applanation tonometry was used to record the augmentation index (Aix) and pulse wave velocity (PWV). We also evaluated plasma levels of C-reactive protein (CRP), Interleukin-1beta (IL-1β), tumor necrosis factor-alpha (TNF-α), Interleukin-6 (IL-6) and Interleukin-10 (IL-10), E-selectin, P-selectin, intercellular adhesion molecule-1 (ICAM-1), vascular cell adhesion molecule-1 (VCAM-1), von Willebrand Factor (vWF) plasma levels, tissue plasminogen activator (TPA) and plasminogen activator inhibitor-1 (PAI-1).

**Results:**

In subjects with acute ischemic stroke and metabolic syndrome we observed higher median plasma values of immuno-inflammatory markers. In acute ischemic stroke patients and metabolic syndrome in relation of each TOAST subtype we observed a more significant positive correlation between PWV and immuno-inflammatory markers.

**Conclusions:**

Stroke subjects with acute ischemic stroke and metabolic syndrome showed a higher degree of immuno-inflammatory and arterial stiffness indexes possibly due to metabolic background of these types of patients that trigger a more intense immune-inflammatory activation irrespective of stroke subtype, whereas being related to stroke subtype in subjects without metabolic syndrome.

## Introduction

Arterial stiffness is increasingly recognized as an important determinant of cardiovascular risk [1,2] and may be directly involved in the process of atherosclerosis [3]. Factors underlying increased arterial stiffness are not completely understood, but both functional and structural alterations in the vessel wall are thought to be important. Acute and chronic inflammation are also associated with endothelial dysfunction [4,5]. More recently, inflammation has been associated with an increased risk of cardiovascular events and the level of C-reactive protein (CRP), a marker of inflammation, predicts outcome in patients with cardiovascular disease [6,7]. Taken together, these data suggest that inflammation may be a key cause of cardiovascular damage.

Although there are some inconsistencies, a number of recent studies have suggested that in a healthy population, there may be a significant relationship between hs-CRP and measures of arterial stiffness [8,9]. Yasmin et al. found hs-CRP to be related to pulse wave velocity (PWV) but not to Augmentation Index (AIx) [10]. In contrast, Kampus et al. found hs-CRP to be independently and significantly associated with AIx but not with the timing of the reflected pressure wave (RT) [8]. However, in inflammatory conditions, the relationship may be stronger. In patients with systemic vasculitis, in which hs-CRP levels are markedly elevated, they were positively correlated with PWV and AIx [9].

Metabolic syndrome is a cluster of common pathologies: abdominal obesity linked to an excess of visceral fat, insulin resistance, dyslipidemia and hypertension [11]. At the molecular level, metabolic syndrome is accompanied by dysregulation in the expression of adipokines (*cytokines and chemokines*) making metabolic syndrome an inflammatory condition. The molecular mechanism underlying the relationship between metabolic syndrome and neurological disorders is not fully understood. However, it is becoming increasingly evident that all cellular and biochemical alterations observed in metabolic syndrome such as impairment of endothelial cell function, abnormality in essential fatty acid metabolism and alterations in lipid mediators along with abnormal insulin/leptin signaling may represent a pathological bridge between metabolic syndrome and neurological disorders such as stroke.

The association of MetS with arterial stiffness has been investigated in many studies [12-14]; however, most of these studies were cross-sectional [14].

In a recent study [15] we reported that stroke patients with metabolic syndrome, compared to stroke subjects without metabolic syndrome, and compared to control subjects without stroke showed a higher mean PWV. We also showed in the same study that PWV value in lacunar subjects with metabolic syndrome was significantly higher compared to value observed in subjects with lacunar stroke without metabolic syndrome. In the same study we reported that PWV in subjects with atherosclerotic and cardioembolic subtypes of stroke and metabolic syndrome was significantly higher compared to values observed in subjects with these stroke subtypes and without metabolic syndrome.

Thus it’s presumable that these findings observed in stroke patients with metabolic syndrome could be due to inflammatory and metabolic background of subjects with metabolic subjects that could influence acute inflammatory activation after an acute ischemic stroke and arterial stiffness process.

On this basis, as an extension of this previous study and on the same sample of patients, we analyzed plasma levels of some immuno-inflammatory markers with the aim to evaluate the relationships between arterial stiffness and immune-inflammatory markers such as cytokines, selectins and adhesion molecules in subjects with acute ischemic stroke with and without metabolic syndrome.

## Materials and methods

### Patient selection

Patients were considered as having metabolic syndrome if they had ≥3 of the following components, based on modification of the NCEP/ATP III: hypertension defined as systolic blood pressure ≥130 mm/Hg and/or a diastolic blood pressure ≥85 mm/Hg, and by history of hypertension, raised plasma triglycerides (≥150 mg/dL [1.7 mmol/L]), low high-density lipoprotein cholesterol levels (≥40 mg/dL in men and ≥50 mg/dL in women), and impaired fasting glucose defined as fasting plasma glucose concentration ≥110 mg/dL (6.1 mmol/L), but less than the criteria for diabetes of ≥125 mg/dL (≥7.0 mmol/L). As a definition of obesity, we used a body mass index ≥30 kg/m2 instead of WHR on a basis of modification of the NCEP/ATP III [16].

We enrolled all consecutive patients with a diagnosis of acute ischemic stroke admitted to the Internal Medicine Department at the University of Palermo between November 2009 and January 2011, and, as controls, hospitalized patients with acute ischemic stroke without a diagnosis of metabolic syndrome and hospitalized patients without a diagnosis of acute ischemic stroke, admitted, in the same period, to our Internal Medicine Department for any cause other than acute cardiovascular and cerebrovascular events.

Stroke was defined by focal neurological signs or symptoms thought to be of vascular origin that persisted for >24 hours, confirmed by brain CT and/or MRI in baseline conditions and brain CT with contrast medium after 48-72 hours [17].

In order to match patients with acute ischemic stroke and controls for cardiovascular risk and previous cardiovascular morbidity, controls were included if they had vascular risk factors or a history of myocardial infarction or cerebro-vascular disease or peripheral vascular disease, but they were excluded if they had either current or recent (within six months) cerebro-vascular disease or one of the exclusion criteria (see above).

Cardiovascular risk factors were evaluated for both cases and controls on the basis of the criteria shown below. Hypercholesterolemia was defined as the presence of total cholesterol blood levels ≥200 mg/dL. Hypertension was defined as present if subjects had been previously diagnosed according to the World Health Organization/International Society of Hypertension guidelines and were routinely receiving antihypertensive therapy.

Patients were defined as type 2 diabetics if they had known diabetes treated by diet, oral hypoglycemic drugs or insulin before stroke.

Previous coronary artery disease was determined on the basis of a history of physician-diagnosed angina, myocardial infarction, or any previous revascularization procedure assessed by a questionnaire.

Previous cerebro-vascular disease (TIA/ischemic stroke) was assessed by history, specific neurologic examination performed by specialists, and hospital or radiological (brain computer tomography or brain magnetic resonance) records of definite previous stroke.

Subjects were classified as having previous peripheral artery disease (PAD) when they had a history of ABI <0.9 and/or of intermittens claudicatio or of critical limb ischemia or when they had undergone a peripheral arterial bypass or amputation.

The study protocol was approved by the Policlinico Universitario “P. Giaccone” Ethical Committee of Palermo, and all participants gave written informed consent. Every subject with ischemic stroke was matched for age (± 3 years), sex, and cardiovascular risk factor prevalence with one control subject.

The type of acute ischemic stroke was classified according to the TOAST classification [18]: 1) Large Artery AtheroSclerosis (LAAS); 2) CardioEmbolic Infarct (CEI); 3) LACunar infarct (LAC); 4) stroke of Other Determined Etiology (ODE); 5) stroke of UnDetermined Etiology (UDE).

All the ischemic stroke patients underwent: medical history with recording of potential stroke risk factors, blood and coagulation tests, 12-lead ECG, 24 hour electrocardiography monitoring, trans-thoracic echocardiography, carotid ultrasound, brain CT or MRI at admission (*repeated between the third and seventh days of stroke onset*).

Neurological deficit score on admission was evaluated by Scandinavian Stroke Scale (SSS). SSS assesses neurological deficit through an evaluation of consciousness level, eye movement, strength in arms, hands, and legs, orientation, language, facial weakness and gait, giving rise to a score ranging from 58 (*absence of deficit*) to 0 (*death*).

### PWV measurement

Carotid-femoral PWV was measured in the supine position using the automatic device (SphygmoCor version 7.1) that measured the time delay between the rapid upstroke of the carotid and femoral artery pulse waves. The distance between the 2 arterial points was measured on the surface of the body using a tape measure. PWV was calculated as the distance traveled by the arterial pulse wave (meters) divided by the time delay between the 2 arterial points (seconds), thus expressed as meters per second. The "distance between the 2 arterial points" was measured using the total distance between the carotid and femoral sites of measurement [19].

### Pulse Wave analysis

Applanation tonometry was used to record radial artery pressure waveform continuously, and mean values of ≥2 screens of pulse waves of good quality were used for analysis. On the basis of the collected data, an averaged radial pressure waveform was generated and a corresponding aortic pressure waveform and BP calculated by the validated transfer function (SphygmoCor version 7.1). The aortic pressure waveform was used to calculate the AIx (difference in height between the first and second systolic peaks expressed as a percentage of pulse pressure (PP).

### Laboratory evaluation

Blood samples were obtained in the non-fasting state. After 10 minutes of rest in the supine position, vital signs were recorded and blood samples were collected from the antecubital vein.

EDTA-anticoagulated peripheral blood was drawn from each patient within 12 hours from symptom onset. Serum and plasma were immediately separated by centrifugation and stored in aliquots at -80°C until analysis.

We evaluated plasma levels of C-reactive protein (CRP), Interleukin-1beta (IL-1β), tumor necrosis factor-alpha (TNF-α), Interleukin-6 (IL-6) and Interleukin-10 (IL-10), E-selectin, P-selectin, intercellular adhesion molecule-1 (ICAM-1), vascular cell adhesion molecule-1 (VCAM-1), as markers of immune-inflammatory activation, von Willebrand Factor (vWF) plasma levels as a marker of endothelial dysfunction, tissue plasminogen activator (TPA) and plasminogen activator inhibitor-1 (PAI-1) as marker of thrombotic/ fibrinolytic pathway.

IL-1β, TNF-α, IL-6 and IL-10 and VWF antigen were measured using a sandwich ELISA (*Human IL-1β, TNF-α, IL-6 and IL-10* Quantikine, R&D Systems *(VWF ELISA kitdurian, Instrumentation Laboratory, Milano, Italy*); VCAM-1, ICAM-1, E-selectin, P-selectin, PAI-1 and TPA-antigen were measured by commercial bioimmunoassay (*Human sICAM-1, sVCAM-I, sE-selectin and sP-selectin Parameter,* Quantikine, R&D Systems, Gentaur AssayMax Human Plasminogen Activator Inhibitor-1 (PAI-1) ELISA Kit, Gentaur AssayMax Tissue Plasminogen Activator (TPA) ELISA Kit).

The minimum detectable concentrations for the diagnostic tests are: TNF alpha: 1.6 pg/mL; IL-1β: <1 pg/mL; IL-6: <0.70 pg/mL; IL-10: >3.9 pg/mL; ICAM-1: < 0.35 ng/mL; VCAM-1: 0.6 ng/mL; E-Selectin: <0.1 ng/mL; P-Selectin: < 0.5 ng/mL; vWF: 1.0%; TPA: 0.3 pg/ml; PAI-1: <50 pg/ml.

Intraassay and interassay coefficients of variation were: TNF alpha: 4.2% and 4.6%; IL-1β: 3.3% and 4.2%; IL-6: 1.6% and 3.3%; IL-10: 4.3% and 7.5%; ICAM-1: 4.8% and 6.1%; VCAM-1: 3.5% and 7.7%; E-Selectin: 4.8% and 5.7%; P-Selectin: 4.9% and 8.8%; vWF: 5% and 10%; TPA: 4.8% and 5%; PAI-1: 5.7% and 8.3%.

### Statistical analysis

Results are expressed as mean ± SD for continuous variables and percentages for categorical data, with *P* ≥ 0.05 considered significant.

Analysis of normality was performed with the Shapiro–Wilk W test.

Non-normally distributed data were logarithmically (Log10) transformed before analysis.

The relationship between immune-inflammatory markers, PWV, AIx, and other parameters was analyzed using nonparametric methods (Spearman p correlations) after correction for age and gender.

## Results

We enrolled 130 patients with acute ischemic stroke and metabolic syndrome, 127 patients with acute ischemic stroke without metabolic syndrome and 120 control subjects matched for age, sex, cardiovascular risk factors and previous cardiovascular morbidity.

In patients with acute ischemic stroke and metabolic syndrome according to the TOAST criteria, the etiology of stroke was: large-artery atherosclerosis (LAAS) in 49 (37.69%) patients, cardioembolism (CEI) in 45 (34.61%) patients, lacunar stroke in 32 (24.61%) patients whereas 3 (2.3%) subjects were classified as ODE and 2 UDE (0.76%).

In patients with acute ischemic stroke without metabolic syndrome, according to the TOAST criteria, the etiology of stroke was large-artery atherosclerosis (LAAS) in 52 (40.94%) patients, cardioembolism (CEI) in 37 (29.7%) patients, lacunar stroke in 35 (27.55%) patients whereas 1 (1.57%) subjects were classified as ODE and 1 (0.78%) as UDE.

Baseline characteristics in stroke patients and in relation to each TOAST subtype are given in Tables 1 and 2.

**Table 1:** Premorbid cardiovascular risk factors, clinical characteristics and medication

**Variable**	**Stroke pts with metabolic syndrome (n: 130)**	**Stroke pts without metabolic syndrome (n: 127)**	**Controls (n: 109)**	***P***
**Age (years)**	64 (61-72.5)	72 (69-83.5)	69 (63-80)	0.031
**M/F (n)**	69/61	60/67	54/55	0.027
**SBP/DBP (mm/Hg)**	152±7.9/98±6.2	147±8.9/92±3.2	141±9.8/91±2.2	<0.001
**diabetes (n/%)**	63 (48.46)	39 (30% )	47 (41.28%)	0.263
**Hypertension (n/%)**	66 (50.79%)	47 (37%)	48 (44.03%)	0.301
**Glucose blood levels (mg/dl)**	149.3 (97-186)	129.1 (88-142)	102 (81.5-133.5)	<0.001
**Cholesterol blood levels (mg/dl)**	241 (189-270)	222 (179-245)	210 (167-225)	<0.001
**Triglyceride blood levels (mg/dl)**	197.5 (149.75-210.75)	157.5 (119.4-181.75)	167.5 (139.4-192.1)	0.004
**White blood cells (per mm³)**	9100 (6200-11000)	8200 (7100-10500	7400 (6500-9800)	<0.001
**Stroke subtype**				
**• LAAS**	49 (37.69)	52 (40.94)		
**• Lacunar**	45 (34.61)	37 (29.7)		
**• CEI**	32 (24.61)	35 (27.55)		
**• ODE**	3 (2.3)	2 (1.57)		
**• UDE**	1 (0.76)	1 (0.78)		
**AIx (%)**	105 ± 3.5	101 ± 4.1	89 ± 4.6	<0.001
**PWV (m/s)**	12.9 ± 3.3	11.2 ± 3.2	10.02 ± 2.29	<0.001
**SSS**	27.12±16.21	29.65±19	30±16.21	
**IL-1-beta (pg/ml)**	10 (6-11)	8 (5-16)	4 (2-5)	<0.001
**IL-6 (pg/ml)**	12 (9-31)	10.3 (7-29)	8 (3.1-12)	<0.001
**TNF-α (pg/ml)**	35.5 (10.25-46)	26 (10.55-49)	5.1 (1.1-4.3)	<0.001
**E-selectin (ng/ml)**	4.05 (2.0-5.8)	2.1 (1.0-3.8)	2 (1-2)	<0.001
**P-selectin (ng/ml)**	6.5 (3-8.9)	4.5 (2-6.8)	3,1 (2.1-4)	0.004
**VICAM-1 (ng/ml)**	18 (12.1-23)	16 (10.1-20)	10 (7-15)	<0.001
**ICAM-1 (ng/ml)**	17.8 (12.2-20)	13.5 (12.2-20)	10.9 (12-16.1)	<0.001
**IL-10 (pg/ml)**	2.05 (2-7)	3.15 (2-7)	5 (2-10)	0.031
**vWF (ng/ml)**	11 (6-15)	4 (3-9)	2 (1-5)	0.0001
**PAI-1 (pg/ml)**	137 (99.5-155)	23 (11-24)	15 ( 7-22)	<0.001
**TPA (pg/ml)**	21 (10.55-39)	55 (29-88)	11 (8-19)	0.44
**CHF (n/%)**	23 (17.69)	20 (15.74)	15 (14.70)	0.065

SBP: systolic blood pressure; DBP: diastolic blood pressure; MAP: mean arterial pressure; AIx: augmentation index; PWV: pulse wave velocity; SSS: Scandinavian Stroke Scale score; NIHSS: National Institutes of Health Stroke Scale; Angiotensin II receptor blockers (ARBs); CAD: coronary artery disease; CHF: congestive heart failure; TIA: transitory ischemic attack; LVH: left ventricular hypertrophy; WMHLS: white matter hypertension lesions.

Demographic and history data are expressed as n° (percentage).

**Table 2:** Laboratory and clinical variables and arterial stiffness markers by ischemic stroke subtype in subjects with ischemic stroke and metabolic syndrome and in patients with acute ischemic stroke without metabolic syndrome

**Variable**	**Lacunar**		***p***	**LAAS**		***p***	**CEI**		***p***
**Number**	**With metabolic syndrome (n: 49)**	**Without metabolic syndrome (n 52)**		**With metabolic syndrome (n: 45)**	**Without metabolic syndrome (n: 37)**		**With metabolic syndrome (n:32)**	**Without metabolic syndrome (n:35)**	
**SBP/DBP (mm/Hg)**	148±6.5/94±4.5	144±45/9235	0.020	146±3.4 /93±4.5	145.5/91±2.9	0.89	144±3.8/ 90±3.1	143±2.8/90±4.1	0.71
**Diabetes (n/%)**	27 (55.10 )	22 (42.30)	0.025	19 (42.22 )	17 (45.94)	0.71	9 ( 28.13)	10 (28.57 )	0.51
**Hypertension (n/%)**	29 (59.18 )	28 (53.84)	0.040	19 (41.30 )	20 (54.05)	0.013	14 ( 43.75)	12 (34.28)	0.011
**Previous stroke (n/%)**	19 (38.77)	14 (26.92)	0.010	16 (34.78)	10 (27.02)	0.041	11 (34.37)	9 (25.71)	0.027
**AIx (%)**	116. 3.5	112 ± 2.8	0.030	111±3.7	108±2.9	0.031	108±4.4	107±3.5	0.021
**PWV (m/sec)**	14.45±2.6	12.81 ±2.1	0.030	11.2 ± 2.31	10.4 ± 2.22	0.030	11.48 ± 1.98	11.25±2,65	0.030
**NIHSS**	17.65±14.59	15.64 ± 8.73	0.021	22.51±16.06	19.00±12.72	0.034	25.23±8.78	22.23±8.78	0.023
**CAD (n/%)**	18 (36.73)	14 (26.92)	0.011	16 (35.5)	12 ( 32.43)	0.71	8 (25)	8 (20)	0.07
**Micro-albuminuria (n/%)**	21 (42.85)	17 (32.76)	0.02	17 (37.77)	9 (24.37)	0.03	9 ( 28.12)	7 (20)	0.045
**Carotid plaque (n/%)**	14 (28.57)	9 (17.30 )	0.031	16 (35.55)	11 ( 29.79 )	0.023	7 (21.87)	5 (14.28 )	0.02
**LVH (n/%)**	11 (22.44)	14 (26.92)	0.021	9 (20)	6 (16.27 )	0.011	10 ( 31.25)	9 (25.71)	0.03
**Previous brain infarct at neuro-imaging**	18 (36.73)	12 (23.07)	0.011	14 (30.43)	9 ( 24.32)	0.021	9 (28.12)	7 (20)	0.02
**WMHLS (n/%)**	19 (38.77)	14 (26.92)	0.021	13 (28.26)	8 (21.62)	0.032	11 (34.37)	8 (22.85 )	0.021
**CRP (mg/dl)**	4.1 ± 2,3	3.5 ± 2.0	0.011	5.0 ± 1.8	3.9 ± 2.1	0.018	5.8 ± 1.6	4.1 ± 1.5	0.020
**IL-1-beta (pg/ml)**	7 (5-11)	5.2 (3-8)	0.012	9 (5-12)	7.9 (4.5-12)	0.017	12 (6-17)	10.8 (6-14)	0.021
**IL-6 (pg/ml)**	9.8 (6,5-19)	8 (6-21)	0.021	12 (6-27)	10.4 (5-23.5)	0.014	13.5 (5-31)	10.2 (4-26)	0.019
**TNF-α (pg/ml)**	29.5 (9.5-33)	23.5 (8. 5-29)	0.025	41.5 (11.25-47)	37.5 (11.6-41)	0.031	43.5 (21-58)	35.5 (20-50)	<0.05
**E-selectin (ng/ml)**	5.05 (1.8-5.7)	3.25 (1.7-5.7)	<0.05	4.85 (1.96-7.9)	2.75 (1.6-5.6)	<0.05	4.15 (2.2-4.1)	3.55 (2.1-4.1)	0.011
**P-selectin (ng/ml)**	4.5 (2-6.8)	3.1 (2-4.7)	0.022	5.4 (2-8.0)	4.3 (2.7-6)	0.034	6.2 (3-8.9)	4.9 (2-7.5)	0.018
**VICAM-1 (ng/ml)**	19 (10.1-21)	15 (8.1-22)	0.031	17.5 (9.2-20)	15 (8.2-19.8)	0.022	19 (10.1-25)	17.8 (11.1-23)	0.041
**ICAM-1 (ng/ml)**	20.8 (15.2-27 )	15.8 (10.2-17 )	<0.05	18.9 (11.2-23)	16.9 (9.8-20)	0.028	22.6 (12.2-29)	21.3 (10-27)	0.021
**IL-10 (pg/ml)**	2.95 (2-7)	3.15 (2-8)	0.45	2.96 (2-7)	3.17 (2-6)	0.52	3.2 (2-9)	23.2 (2-9)	0.39
**vWF (ng/ml)**	12 (4-19)	9 (4-16)	<0.05	14 (5-19)	11 (5-16)	<0.05	14 (6-18)	12 (6-18)	0.023
**PAI-1 (pg/ml)**	145 (97.5-178)	137 (98.5-149)	<0.05	139 (79.4-165)	128(91.5-135)	<0.05	139 (99.0-145)	109 (99.0-145)	<0.05
**TPA (pg/ml)**	28 (16.15-39)	24 (12.15-35)	0.021	26.7 (11.4-31)	22 (10.5-29)	0.031	24 (12.1-47)	20 (10.1-39)	0.021

SBP: systolic blood pressure; DBP: diastolic blood pressure; MAP: mean arterial pressure; AIx: augmentation index; PWV: pulse wave velocity; SSS: Scandinavian Stroke Scale score; NIHSS: National Institutes of Health Stroke Scale ; Angiotensin II receptor blockers (ARBs); CAD: coronary artery disease; CHF: congestive heart failure; LVH: left ventricular hypertrophy; WMHLS: white matter hypertension lesions.

Demographic and history data are expressed as n° (percentage).

In subjects with acute ischemic stroke and metabolic syndrome compared to subjects with acute ischemic stroke without metabolic syndrome and control subjects we observed higher median plasma values of IL-1-beta [10 (6–11) pg/ml) vs. 8 (5–16) pg/ml vs. 4 (2–5) pg/ml; p < 0.001]; IL-6 [12 (9–31) pg/ml) vs. 10.3 (7–29) pg/ml vs. 8 (3.1-12) pg/ml; p < 0.001]; TNF-α 35.5 (10.25-46) pg/ml) vs. 26 (10.55-49) pg/ml vs. 5.1 (1.1-4.3) pg/ml; p < 0.001]; E-selectin [4.05 (2.0-5.8) ng/ml) vs. 2.1 (1.0-3.8) ng/ml vs. 2 (1, 2) ng/ml; p < 0.001]; P-selectin [6.5 (3-8.9) ng/ml) vs. 4.5 (2-6.8) ng/ml vs. 3,1 (2.1-4) ng/ml; p < 0.001]; VICAM-1 [18 (12.1-23) ng/ml) vs. 16 (10.1-20) ng/ml vs. 10 (7–15) ng/ml; p < 0.001]; ICAM-1 [17.8 (12.2-20) ng/ml) vs. 13.5 (12.2-20) ng/ml vs. 10.9 (12-16.1) ng/ml; p < 0.001]; vWF [17.8 (12.2-20) ng/ml) vs. 13.5 (12.2-20)ng/ml vs. 10.9 (12-16.1) ng/ml; p < 0.001]; PAI-1 [137 (99.5-155) vs. 23(11–24) ng/ml vs. 10.9 15 (7–22) and lower median plasma values of IL-10 [ 11 (6–15) pg/ml) vs. 4 (3–9) pg/ml vs. 2 (1–5) pg/ml; p < 0.001] ( see Table 1).

Among subjects with acute ischemic stroke we observed higher plasma values of CRP, IL-1β, IL-6, TNF-α, E-Selectin, P-Selectin, ICAM-1, VCAM-1, wWF, PAI-1 in subjects with LAAS, lacunar and CEI subtype ischemic stroke and metabolic syndrome compared to subjects with this subtype of stroke without metabolic syndrome (see Table 2).

### Relationship between PWV and immune-inflammatory variables

In subjects with acute ischemic stroke and metabolic syndrome compared to subjects with acute ischemic stroke without metabolic syndrome we observed a more significant positive correlation for age, and gender, between PWV and CRP (r = 0.38 vs. r =0.31; p = 0.021); TNF-α (*r* = 0.44 vs. r = 0.34 *p* < 0.05), IL1β (*r* = 0.39 vs. r = 0.30; *p*<<0.05), IL-6 (*r* = 0.42 vs. r = 0.35 *p* = 0.032), E-selectin (*r* = 0.36 vs. r = 0.29; *p* = 0.038), P-selectin (*r* = 0.34 vs. r = 0.28; *p* = 0.041), and IL-6 (*r* = 0.21; *P* < 0.05), vWF (*r* = 0.40 vs. r = 0.34; *p* = 0.019), PAI-1 (r = 0.37 vs. r = 0.29; *p*<0.05) (see Table 3).

**Table 3:** Correlations of PWv and Aix with immune-inflammatory variables

**Variable**	**Pulse wave velocity**			**Augmentation Index**		
**Number**	**With metabolic syndrome**	**Without metabolic syndrome**	***p***	**With metabolic syndrome**	**Without metabolic syndrome**	***p***
	**(n: 49)**	**(n: 52)**		**(n: 45)**	**(n: 37)**	
**CRP**	**0.38**	**0.31**	**0.021**	**0.27**	**0.26**	0.73
**IL-1-β**	**0.39**	**0.30**	**<0.05**	**0.21**	**0.23**	0.66
**IL-6**	**0.42**	**0.35**	**0.032**	**0.33**	**0.23**	**0.022**
**TNF-α**	**0.44**	**0.34**	**<0.05**	**0.32**	**0.26**	**0.033**
E-selectin	**0.36**	**0.29**	**0.038**	**0.19**	**0.21**	0.56
P-selectin	**0.34**	**0.28**	**0.041**	**0.18**	**0.16**	0.44
VICAM-1	**0.30**	**0.29**	0.70	**0.20**	**0.22**	0.66
ICAM-1	**0.30**	**0.29**	0.81	**0.22**	**0.24**	0.63
IL-10	**0.19**	**0. 18**	0.42	**0.22**	**0. 23**	0.56
**vWF**	**0.40**	**0.34**	**0.019**	**0.36**	**0.31**	**0.031**
PAI-1	**0.37**	**0.29**	**<0.05**	**0.18**	**0.15**	0.77
TPA	**0.33**	**0.25**	**0.022**	**0.20**	**0.21**	0.69

Coefficients (*r*) and *p*-values are calculated by the Pearson correlation mode.

SBP: systolic blood pressure ; DBP: diastolic blood pressure; MAP: mean arterial pressure; AIx: augmentation index; PWV: pulse wave velocity; SSS: Scandinavian Stroke Scale score; NIHSS: National Institutes of Health Stroke Scale; Angiotensin II receptor blockers (ARBs); CAD: coronary artery disease ; CHF: congestive heart failure; LVH: left ventricular hypertrophy; WMHLS: white matter hypertension lesions; TIA: transitory ischemic attack; SSS: Scandinavian Stroke Scale score; NIHSS: National Institutes of Health Stroke Scale;

Demographic and history data are expressed as n° (percentage).

Significant *R* and *p* are in bold.

### Relationship between AIx and and immune-inflammatory variables

In subjects with acute ischemic stroke and metabolic syndrome compared to subjects with acute ischemic stroke without metabolic syndrome we observed a more significant positive correlation for age, and gender, between AIX and IL-6 (r = 0.33 vs. r =0.23; p = 0.022), TNF-α (r =0.32 vs. r = 0.26; p = 0.033) and vWF (r = 0.36 vs. r =0.31; p = 0.031) (see Table 3).

### Relationship Between PWV and immune-inflammatory variables in relation to TOAST subtype

Among subjects with lacunar subtype and metabolic syndrome, PWV (after correction for age and gender) was more significantly and positively related to CRP, IL-1β, IL-6, TNF-α, vWF and PAI-1 (see Table 4) compared to subjects without metabolic syndrome and the same subtype of stroke.

**Table 4:** Correlations of PWv with clinical variables and cardiovascular risk factors in relation to TOAST stroke subtype

	**Lacunar**			**LAAS**			**CEI**		
**Variable**	**Pulse wave velocity (PWv)**			**Pulse wave velocity (PWv)**			**Pulse wave velocity (PWv)**		
	**With metabolic syndrome (n: 49)**	**Without metabolic syndrome (n 52)**	***p***	**With metabolic syndrome (n: 45)**	**Without metabolic syndrome (n: 37)**	***p***	**With metabolic syndrome (n:32)**	**Without metabolic syndrome (n:35)**	***p***
**CRP**	**0.32**	**0.27**	**0.031**	**0.38**	**0.32**	**0.041**	**0.29**	**0.28**	**0.63**
**IL-1-β**	**0.34**	**0.29**	**0.029**	**0.37**	**0.29**	**<0.05**	**0.31**	**0.28**	**0.75**
**IL-6**	**0.33**	**0.28**	**0.030**	**0.35**	**0.32**	**0.041**	**0.29**	**0.28**	**0.63**
**TNF-α**	**0.32**	**0.27**	**0.028**	**0.31**	**0.29**	**<0.05**	**0.31**	**0.28**	**0.75**
E-selectin	**0.32**	**0.23**	**0.65**	**0.31**	**0.23**	**0.041**	**0.19**	**0.20**	0.61
P-selectin	**0.34**	**0.27**	**0.071**	**0.29**	**0.24**	**<0.05**	**0.17**	**0.21**	0.37
VICAM-1	**0.33**	**0.29**	**0.060**	**0.36**	**0.26**	**0.041**	**0.19**	**0.20**	0.61
ICAM-1	**0.34**	**0.26**	**0.072**	**0.38**	**0.33**	**0.041**	**0.17**	**0.21**	0.37
IL-10	**0.20**	**0.22**	**0.56**	**0.21**	**0.23**	**0.66**	0.23	0.25	0.57
**vWF**	**0.37**	**0.31**	**0.023**	**0.41**	**0.34**	**<0.05**	0.40	0.31	**<0.05**
PAI-1	**0.31**	**0.22**	**0.043**	**0.34**	**0.23**	**0.041**	**0.19**	**0.20**	0.61
TPA	**0.29**	**0.26**	**0.55**	**0.26**	**0.24**	**0.75**	**0.20**	**0.21**	0.37

Coefficients (*r*) and *p*-values are calculated by the Pearson correlation mode.

SBP: systolic blood pressure; DBP: diastolic blood pressure; MAP: mean arterial pressure; AIx: augmentation index; PWV: pulse wave velocity; SSS: Scandinavian Stroke Scale score; NIHSS: National Institutes of Health Stroke Scale; Angiotensin II receptor blockers (ARBs); CAD: coronary artery disease; CHF: congestive heart failure; LVH: left ventricular hypertrophy; WMHLS: white matter hypertension lesions.

Demographic and history data are expressed as n° (percentage).

Significant *R* and *p* are in bold.

In subjects with stroke classified as LAAS and metabolic syndrome, PWV was more significantly and positively related to CRP, IL-1β, IL-6, TNF-α, vWF and PAI-1 (see Table 4) compared to subjects without metabolic syndrome and the same subtype of stroke.

In subjects with CEI subtype and metabolic syndrome, PWV was more significantly and positively related to vWF but not with TNF-α, CRP, IL-6, IL-1β and PAI-1 (see Table 4) compared to subjects without metabolic syndrome and the same subtype of stroke.

### Relationship between AIx and immune-inflammatory variables in relation to TOAST subtype

Among subjects with Lacunar subtype, AIx was more positively and significantly correlated (after correction for age and gender) only to TNF-α, IL-1β and vWF compared to subjects without metabolic syndrome; in subjects with LAAS subtype and metabolic syndrome we observed a more significant and positive relationship between AIx and IL-6. vWF and TNF-α compared to subjects without metabolic syndrome; in CEI group with metabolic syndrome IL-6, TNF-α and v-WF were more positively and significantly related to AIx compared to subjects without metabolic syndrome (see Table 5).

**Table 5:** Correlations of AIx with clinical variables and cardiovascular risk factors in relation to TOAST stroke subtype

	**Lacunar**			**LAAS**			**CEI**		
**Variable**	**Augmentation Index (AIx)**		***p***	**Augmentation Index (AIx)**		***p***	**Augmentation Index (AIx)**		***p***
	**With metabolic syndrome (n: 45)**	**Without metabolic syndrome (n: 37)**	**0**	**With metabolic syndrome (n: 45)**	**Without metabolic syndrome (n: 37)**		**With metabolic syndrome (n:32)**	**Without metabolic syndrome (n:35)**	
**CRP**	**0.24**	**0.26**	0.73	0.27	0.26	0.81	0.20	0.21	0.73
**IL-1-β**	**0.21**	**0.22**	0.60	0.24	0.25	0.66	0.20	0.22	0.56
**IL-6**	**0.31**	**0.25**	**0.026**	**0.34**	**0.226**	**0.011**	**0.33**	**0.23**	**0.022**
**TNF-α**	**0.33**	**0.27**	**0.022**	**0.32**	**0.26**	0.033	**0.32**	**0.26**	**0.033**
E-selectin	**0.20**	**0.27**	0.56	0.19	0.21	0.56	0.19	0.21	0.56
P-selectin	**0.19**	**0.15**	0.33	0.18	0.16	0.44	0.18	0.16	0.44
VICAM-1	**0.21**	**0.22**	0.66	0.22	0.21	0.66	0.20	0.22	0.66
ICAM-1	**0.22**	**0.24**	0.63	0.20	0.24	0.63	0.22	0.24	0.63
IL-10	**0.22**	**0. 23**	0.56	0.22	0. 23	0.56	0.22	0. 23	0.56
**vWF**	**0.36**	**0.30**	**0.020**	**0.33**	**0.27**	**0.029**	**0.36**	**0.32**	**0.018**
PAI-1	**0.19**	**0.14**	0.64	0.16	0.14	0.77	0.18	0.15	0.77
TPA	**0.22**	**0.25**	0.77	0.20	0.21	0.69	0.20	0.21	0.69
									<0.05
									0.033

Coefficients (*r*) and *p*-values are calculated by the Pearson correlation mode.

SBP: systolic blood pressure; DBP: diastolic blood pressure; MAP: mean arterial pressure; AIx: augmentation index; PWV: pulse wave velocity; SSS: Scandinavian Stroke Scale score; NIHSS: National Institutes of Health Stroke Scale ; Angiotensin II receptor blockers (ARBs); CAD: coronary artery disease; CHF: congestive heart failure; LVH: left ventricular hypertrophy; WMHLS: white matter hypertension lesions. Demographic and history data are expressed as n° (percentage). Significant *R* and *p* are in bold.

## Discussion

Our main findings were that patients with acute ischemic stroke and metabolic syndrome had both increased inflammatory markers and arterial stiffness indexes, compared to control subjects with acute ischemic stroke without metabolic syndrome.

This is the first study, to the best of our knowledge, to show in patients with acute ischemic stroke and metabolic syndrome that circulating levels of some immune-inflammatory markers such as CRP, IL-6, IL-1 β, TNF-α and VWF are more significantly correlated to PWV and wave reflection compared to subjects without metabolic syndrome.

The existence of a strong association between the presence of MetS and arterial stiffness has been shown in many cross-sectional studies [20,21].

Inflammation is an important component of metabolic syndrome (MetS) which could be the link between the metabolic and cardiovascular consequences of this condition. Low-grade systemic inflammation, elevated leptin concentration and low adiponectin level are described in very young obese children, correlating with a range of variables of metabolic syndrome. Inflammation and adipocytokines can play an important role in the etiology of metabolic syndrome [21].

Results from some studies [20,21] show that adiponectin concentrations are reduced with increased adiposity, while others show that TNF-α, IL-6, resistin and CRP concentrations may be elevated with increased adiposity. A possible link exist between decreased adiponectin with increased insulin resistance, while some evidence links increased TNF-α and resistin with increased insulin resistance Another study relate higher blood pressures to decreased adiponectin, increased TNF-α, and CRP concentrations [22].

Our findings, reporting higher plasma values of immune-inflammatory variables in subjects with acute ischemic stroke and metabolic syndrome, and more significant correlations between these immune-inflammatory variables and arterial stiffness markers in subjects with metabolic syndrome, underlines the role of inflammation in the vascular complications of metabolic syndrome.

Our findings show that both aortic stiffness and wave reflection are more related to the degree of systemic inflammation in stroke subjects with metabolic syndrome, suggesting that circulating inflammation mediators such as CRP and some pro-inflammatory cytokines can influence the stiffness of vessels distant to those involved in the disease process itself.

This finding also emphasizes the systemic nature of the inflammatory response both in the atherosclerosis process and after acute ischemic stroke, encompassing biochemical and hemodynamic parameters. Cytokine plasma levels in their turn also influence vascular vulnerability to the inflammatory response and decreased production of the endogenous vasodilator nitric oxide (NO) [23], and it has been demonstrated that inhibition of basal NO synthesis increases aortic AIx75 and velocity in vivo [23].

An immune-inflammatory cascade occurs after an acute ischemic stroke [22,24]*,* so, on this basis, cytokine levels that we reported and measured 72 hr from symptom onset, express an acute rise in these inflammatory markers. What is the relationship between acute high levels of cytokines and arterial stiffness indexes in stroke patients in relation to the presence of metabolic syndrome? Does metabolic syndrome trigger a higher degree of hyper-acute inflammatory state after ischemic stroke directly causing an increase of arterial stiffness? Or in stroke-prone patients with a chronic inflammatory activation due to their metabolic background (*insulin resistance, adipocite dysfunction*) exacerbated by acute stressor events such an acute cerebrovascular event, does inflammation represent the pathogenic basis of higher arterial stiffness indexes levels in patients with acute ischemic stroke and metabolic syndrome compared to stroke patients without metabolic syndrome?

It’s difficult to answer, maybe the peculiar metabolic and inflammatory background of metabolic syndrome may explain the observed relationship between immune-inflammatory markers, arterial stiffness indexes and acute ischemic stroke in metabolic syndrome patients.

In diabetes and metabolic syndrome exists a complex interrelationship of various inflammatory variables with metabolic disorders and their effect on the cardiovascular system. A simplified explanation can be that inflammation increases insulin resistance, which in turn leads to obesity while perpetuating diabetes, high blood pressure, prothrombotic state and dyslipidaemia [25]. Some studies [26,27] have produced data suggesting an interplay between hormones, cytokines and resistin. Insulin resistance represents a common metabolic abnormality leading to cardiovascular disease and cerebrovascular complications. As several cytokines are also produced by adipose tissue [28] it was postulated that an “adipo-vascular” axis [29] may contribute to the increased risk of cardiovascular events in patients with metabolic syndrome. In patients with metabolic syndrome this “adipo-vascular axis" expresses itself in lower plasma levels of adiponectin and higher plasma levels of IL-6 that could be linked to the development of arterial stiffness and to the pathogenesis of ischemic stroke through microvascular and inflammatory mechanisms. Inflammation has been emphasized as a critical factor in the pathogenesis and destabilization of atherosclerotic lesions [30]. Early in the course of diabetes and metabolic syndrome intracellular hyperglycemia due to insulin resistance causes changes in blood flow and increased vascular permeability. This reflects decreased activity of nitric oxide, and increased activity of angiotensin II and endothelin-1 [31,32]. Angiotensin II produces acute vasoconstriction, leading to an increase in blood pressure. Endothelin-1 is also an important regulator of vascular tone and has been implicated in the pathogenesis of atherosclerosis. In addition, abnormalities of extracellular matrix contribute to an irreversible increase in vascular permeability. Hyperglycemia itself leads not only to decreased endothelial production of nitric oxide, which represents an anti-atherogenic molecule, but also to increased production of a potent inhibitor of fibrinolysis, namely plasminogen activator inhibitor 1 (PAI-1) [33].

Circulating inflammatory markers, such as hs-CRP, are known to be associated with cardiovascular disease and are useful for risk stratification of patients with cardiovascular disease [34,35]. However, circulating inflammatory markers cannot provide information on the vascular inflammation of local individual vascular lesions.

In subjects with acute ischemic stroke and metabolic syndrome we observed higher plasma values of immune-inflammatory markers in subjects with LAAS, lacunar and CEI subtype of ischemic stroke and metabolic syndrome compared to subjects with these subtypes of stroke without metabolic syndrome. This finding appears especially interesting in relation to the previous findings by our group [36-40] showing a higher degree of immune-inflammatory activation of the acute phase of stroke only in CEI subtype of stroke, whereas in patients with acute ischemic stroke and metabolic syndrome these three subtypes of stroke are characterized by a higher degree of immune-inflammatory activation compared to patients with acute ischemic stroke and without metabolic syndrome. It is as if metabolic background could trigger a high inflammatory cascade irrespective of stroke subtype and of its pathogenesis.

Nevertheless, it’s possible to obtain an answer to the question about the relationship between inflammation markers and arterial stiffness indexes in acute ischemic stroke in subjects with metabolic syndrome by evaluating our findings of some significant correlations at intra-group analysis in each TOAST subtype between arterial stiffness indexes and immune-inflammatory markers. We observed a higher correlation between immune-inflammatory variables and arterial stiffness indexes in subjects with acute ischemic stroke and metabolic syndrome, whereas a subtype oriented analysis showed a higher relationship between inflammatory variables and PWV in LAAS, lacunar and CEI subtypes of stroke. In a previous study [36] by our group cardioembolic stroke appears as the “less atherosclerotic” among each diagnostic subtype of stroke, reporting the correlation of PWV only with CRP and vWF. In our subjects with acute ischemic stroke and metabolic syndrome, metabolic background and inflammatory pathways of these types of patients trigger a more intense immune-inflammatory activation irrespective of stroke subtype and this finding could appear original owing to the fact that no study to our knowledge evaluated inflammatory degree in patients with metabolic syndrome and an acute ischemic event such as ischemic stroke.

In conclusion, the results of our study support the view that patients with metabolic syndrome and acute ischemic stroke have a higher degree of immune-inflammatory markers strictly related to values of arterial stiffness indexes compared to stroke controls without metabolic syndrome and independently of stroke subtypes.

## Abbreviations

SBP: Systolic blood pressure; DBP: Diastolic blood pressure; MAP: Mean arterial pressure; AIx: Augmentation index; PWV: Pulse wave velocity; SSS: Scandinavian Stroke Scale score; NIHSS: National Institutes of Health Stroke Scale; ARBs: Angiotensin II receptor blockers; CAD: Coronary artery disease; CHF: Congestive heart failure; TIA: Transitory ischemic attack; LVH: Left ventricular hypertrophy; WMHLS: White matter hypertensity lesions; VICAM-1: Vascular cell adhesion protein 1; ICAM-1: Intercellular Adhesion Molecule 1; IL-6: Interleukin-6; IL-1 beta: Interleukin 1 beta; IL-10: Interleukin-10; vWF: Von Willebrand Factor; PAI-1: Plasminogen Activator Inhibitor 1; TPA: Tissue plasminogen activator.

## Competing interests

We have no conflict of interest or finantial disclosure.

## Authors’ contributions

Study design AT, RP, GL, AP. Acquisition of data: AT, RP, BC, RD, VA, CB, VD, CM. Analysis and interpretation of data: AT, DD, RP, GL, AP. Manuscript preparation: AT, RP. All authors read and approved the final manuscript.
